# Carbon Dioxide Emissions from Acid Mine Drainage Neutralization Are a Major Component of Metal Mining Carbon Footprints

**DOI:** 10.1021/acs.est.6c05684

**Published:** 2026-07-22

**Authors:** Stanisław Kołodziejski, Eva E. Stüeken, Laura Sánchez López, Jose Miguel Nieto, Carlos R. Cánovas, Rafael Pérez-López, Luke Bridgestock

**Affiliations:** † School of Earth and Environmental Sciences, 7486 University of St Andrews, St Andrews KY16 9TS, U.K.; ‡ Department of Earth Sciences & Research Center on Natural Resources, Health and the Environment, 83150 University of Huelva, Huelva 21071, Spain

**Keywords:** acid mine drainage, CO_2_ emissions, metal mining, carbon footprints, estuarine chemistry

## Abstract

Achieving the transition to net zero human greenhouse gas emissions requires a large increase in metal production by mining. Acid mine drainage (AMD) neutralization is a potentially important but poorly quantified source of mining industry CO_2_ emissions. Here, we show that AMD neutralization-related CO_2_ emissions can be a major component of metal production carbon footprints. Data from the rivers and estuarine system draining the central and eastern Iberian Pyrite Belt show that AMD neutralization emits 32 ± 11 kt CO_2_/yr. Normalized to historic copper production rates, AMD-associated emissions (0.3–4.2 t CO_2_ per t Cu) are of similar magnitude as conventional copper production carbon footprints (1–9 t CO_2_ per t Cu). However, continual oxidative weathering of waste sulfide minerals will increase AMD-related CO_2_ emission budgets into the future. Complete oxidative weathering of waste sulfide minerals extracted from this region will yield AMD-related CO_2_ emissions (7–165 t CO_2_ per t Cu) that exceed conventional Cu production carbon footprints by more than 1 order of magnitude. These findings highlight the need to account for AMD-related CO_2_ emissions from metal mining to support the transition toward net zero greenhouse gas emissions.

## Graphical abstract

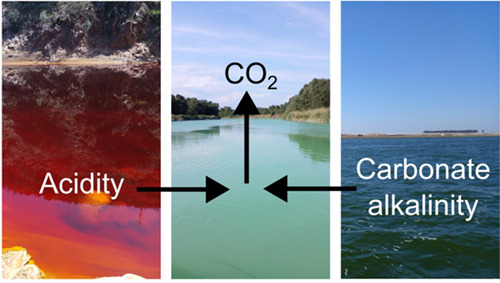

## Introduction

The technology and infrastructure needed to achieve the transition to net zero greenhouse gas emissions require large increases in metal production by mining. Metal mining and production accounts for ∼ 10% of the global energy-related greenhouse gas emissions.^1^ Decarbonizing the metal mining and production industry is, therefore, essential for reaching net zero.^1^ Achieving this requires a comprehensive understanding of the magnitude and nature of the associated CO_2_ emission sources. The neutralization of acid mine drainage (AMD) by carbonate alkalinity is a potentially important, but poorly constrained, mining industry CO_2_ emission source.^1–3^ Here, we show that CO_2_ emissions from AMD neutralization can dominate metal production carbon footprints of sulfide-rich ores.

Acid mine drainage refers to waters with low pH and high dissolved metal concentrations that drain mining regions.^4,5^ This chemistry is produced by the oxidative weathering of metal sulfide minerals, driven by mining activities that expose sulfide minerals within metal ore deposits to oxygenated surface waters.^5–7^ As these waters transit through the environment, their acidity can be neutralized by carbonate alkalinity stored in carbonate minerals or seawater, resulting in CO_2_ emissions to the atmosphere^2,3^ (eq 1).

$$2H^{+}+{\mathrm{CO}}_{3}^{2-}\to H^{+}+{\mathrm{HCO}}_{3}^{-}\to H_{2}O+CO_{2}$$ 1

Despite the recognized potential importance of AMD neutralization reactions as a CO_2_ emission source, their exact contribution to metal mining carbon footprints is poorly known.^1^ A key challenge is that CO_2_ emissions from AMD neutralization tend to be both spatially and temporally decoupled from those of the associated mining processes. They can occur along water flow pathways downstream from mine sites and be spread over decades to millennia following ore extraction and processing due to the kinetics of sulfide mineral oxidation reactions.^8^ Previous studies have largely focused on quantifying the magnitude of CO_2_ emissions from this process associated with coal mining operations.^3,9^ However, those associated with metal mining operations remain poorly constrained. This comes despite the fact that metal ore deposits can be predominantly composed of metal sulfide minerals (massive sulfides; ore featuring 50–100 wt % sulfide minerals^10,11^ ), resulting in high AMD production rates.

This study quantifies the CO_2_ emissions related to AMD neutralization in two of the most heavily AMD-impacted river catchments in the world: the Tinto and Odiel (Spain) (Figure 1). These rivers drain the eastern and central parts of the Iberian Pyrite Belt (IPB), a geological formation that features the highest concentration of volcanogenic massive sulfide (VMS) deposits in the world.^10,11^ This region hosts more than 1600 Mt of massive sulfides, with 82 inactive or working mines, the majority of which reside within the catchment areas of the Odiel and Tinto rivers.^11,12^ These ore bodies have been mined principally for Cu and Zn and typically feature pyrite (FeS_2_) concentrations of ca. 50–100 wt % with Cu grades of 0.4–5 wt %.^11,13^ Mining of these deposits dates to antiquity, but most extraction has occurred since 1875.^12^ It is estimated that 245 Mt of ore was excavated from this region between late 1873 and 2001.^14^ The oxidative weathering of the extracted waste sulfide minerals has rendered the Odiel and Tinto rivers among the most acidic (pH ∼ 2–4) and metal-contaminated rivers on Earth.^4,15,16^ These river catchments therefore represent an endmember case study for constraining the upper magnitude of metal mining CO_2_ emissions from AMD neutralization.

**Figure 1:**
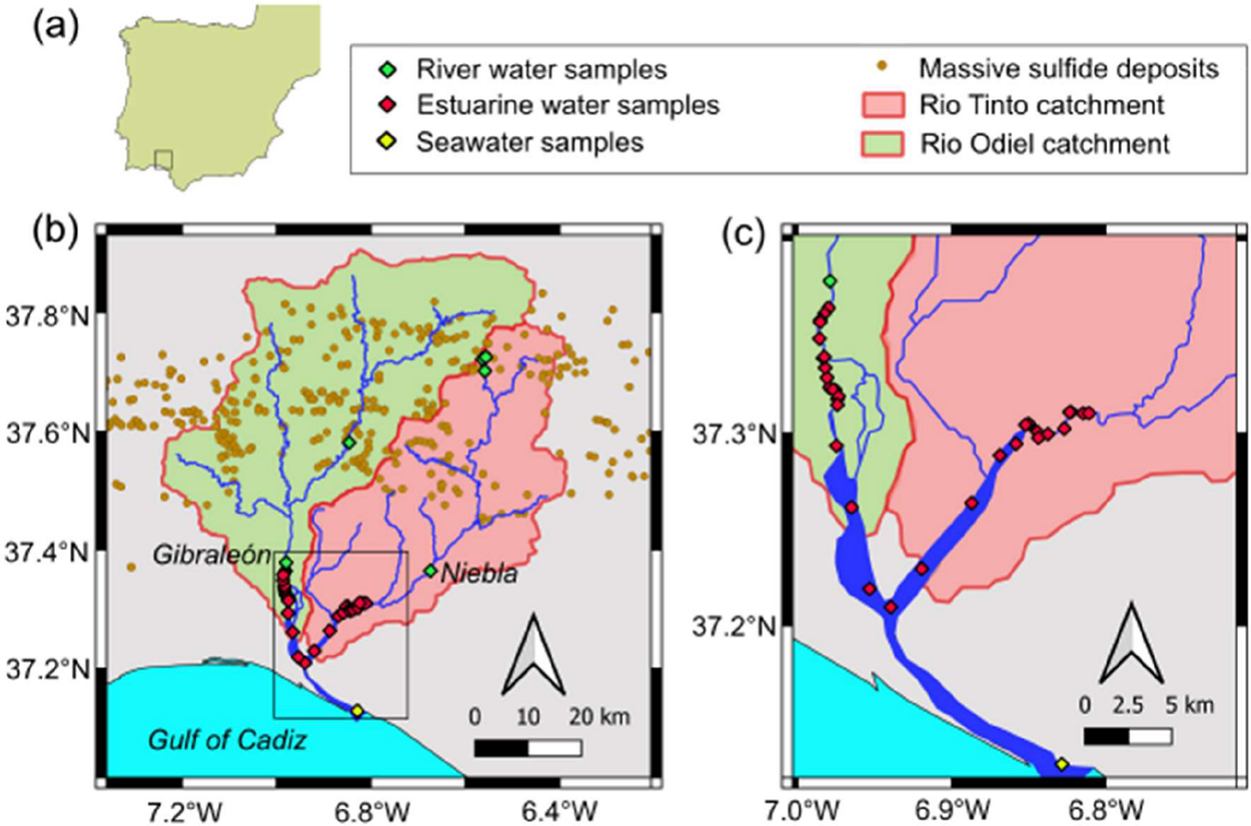
Sample locations within the Odiel and Tinto river catchments. Panel (a) shows an outline of the Iberian Peninsula, with the black box denoting the location of the study area. Panel (b) shows the full catchment area of both rivers, with locations of the water samples measured in this study (diamond symbols) in relation to the drainage network and massive sulfide deposits of the central and eastern Iberian Pyrite Belt (circle symbols). Panel (c) shows the location of water samples from the Ria de Huelva estuary influenced by river–seawater mixing. The black box in panel (b) denotes the area covered in panel (c). Catchment boundaries and river network were sourced from HydroSHEDS.^33^

The magnitude of CO_2_ emissions resulting from AMD neutralization is dependent on the flux of AMD acidity produced and the pathways by which it is neutralized. The acidity of AMD comprises both free H^+^ (as measured by pH) and acidity-producing metal species, principally dissolved Fe^2+^, Fe^3+^, Al^3+^, and Mn^2+^.^17,18^ At low pH, these metals are highly soluble, but as the pH rises during neutralization, they precipitate from solution as oxyhydroxide and oxyhydroxysulfate phases^19,20^ (Supporting Information S.1). These reactions release H^+^ ions equivalent to the positive charge of these metals, buffering the neutralization process.^19^ The acidity of AMD-impacted waters can therefore be approximated as the total positive charge in solution supported by H^+^ and these metal species (eq 2,^17^).

$$\mathrm{Acidity}\left(\mathrm{mM}\right)=\left(2\left[{\mathrm{Fe}}^{2+}\right]+3\left[{\mathrm{Fe}}^{3+}\right]+3\left[{\mathrm{Al}}^{3+}\right]+2\left[{\mathrm{Mn}}^{2+}\right]+1000\left(10^{-\mathrm{pH}}\right)\right)$$ 2

where [X] denotes the concentrations of acidic metal cations in mM. We note that this approximation is impacted by uncertainty in Fe hydrolysis and aspects of S cycling in these systems.^17^ In particular, the precipitation of metal oxyhydroxysulfate phases removes SO_4_^2–^ from solution, lowering the production of H^+^ per unit of acidic metal charge assumed by eq 2.^21,22^

Neutralization of AMD acidity can occur via leaching of base cations (Ca^2+^, Mg^2+^, Na^+^, and K^+^) from silicate and carbonate minerals within the river catchments. Weathering of silicate minerals by AMD acidity features no net CO_2_ emission. Weathering of carbonate minerals by AMD acidity, however, will emit 0.5 mol of CO_2_ per mol of AMD acidity consumed.^23^ Alternatively, AMD acidity can be neutralized by seawater alkalinity upon discharge to the ocean. This neutralization pathway will produce ∼0.9 mol of CO_2_ per mol of AMD acidity, with the exact value depending on the speciation of carbonate alkalinity between HCO_3_^–^ and CO_3_^2–^ in the local seawater (eq 1).

The Odiel and Tinto rivers have been well studied with respect to fluxes of AMD acidity, with multiannual time series of acidic metal fluxes spanning a period of 36 years.^24–29^ Due to the low acid-neutralizing capacity of the underlying geology, dominated by siliciclastic sediments and volcanic rocks with limited carbonates, in-catchment chemical weathering processes do not fully neutralize the acidity of these rivers.^30^ A large fraction of the acidity transported by these rivers is instead neutralized by seawater mixing in the Ria de Huelva estuary.^19,20,31,32^ However, quantitative constraints on the relative importance of different AMD neutralization pathways and their resulting CO_2_ emissions are lacking. These knowledge gaps are addressed using (1) major ionic compositions of Odiel and Tinto river water to constrain in-catchment neutralization pathways and (2) geochemical distributions across the Ria de Huelva estuary and river–seawater mixing experiments to constrain CO_2_ emissions arising from estuarine mixing.

## Materials and Methods

### Constraining the Relative Importance of AMD Neutralization Pathways

Dissolved ionic compositions of Odiel and Tinto river waters collected upstream from the Ria de Huelva estuary (Figure 1) were used to partition AMD neutralization between in-catchment (silicate and carbonate weathering) and estuarine pathways. Samples were taken under two different hydrological conditions: high flow (Odiel: 11.7 m^3^/s; Tinto: 14.6 m^3^/s) in April 2024 and low flow (Odiel: 3.65 m^3^/s; Tinto: 0 m^3^/s) in June 2024. Note that while the gauging station registered a discharge of 0 m^3^/s for the Tinto river in June, visual inspection of the river at this time revealed a small discharge, likely ∼0.001 m^3^/s. Samples were analyzed for pH and dissolved (<0.22 μm) ionic compositions (Fe^2+^, Fe^3+^, Al^3+^, Mn^2+^, Ca^2+^, Mg^2+^, Na^+^, K^+^, SO_4_^2–^, and Cl^–^). Due to the lack of evaporite rocks in the catchments,^34^ it is assumed that SO_4_^2–^ and Cl^–^ are derived solely from sulfide mineral oxidation and sea salt deposition via rainwater, respectively, while the base cations (Ca^2+^, Mg^2+^, Na^+^, and K^+^) are potentially derived from a mixture of sea salt deposition, carbonate, and silicate weathering.^35^ Base cation budgets were corrected for rainfall inputs using the Cl^–^ concentrations and the cation/Cl ratios of seawater.^35^ The relative contributions of AMD neutralization by in-catchment versus estuarine processes were quantified as the ratio of acidity to total cationic charge in solution (eq 3):

$$F_{\mathrm{es}}=\frac{\mathrm{Acidity}}{\mathrm{Acidity}+(2{\mathrm{Ca}}^{2+}+2{\mathrm{Mg}}^{2+}+{\mathrm{Na}}^{+}+K^{+})}$$ 3

where *F*_es_ is the fraction of AMD acidity that will be neutralized by estuarine mixing, acidity is defined by eq 2, and base cations (Ca^2+^, Mg^2+^, Na^+^, and K^+^) are concentrations in mM corrected for rain inputs. The importance of silicate vs carbonate weathering reactions during AMD neutralization within the catchment is assessed by comparing ratios of Ca/Na and Mg/Na corrected for sea salt inputs to those of local rock endmembers.^35,36^

### Carbon Dioxide Emission Estimates Based on Estuarine Geochemical Distributions

Estuarine distributions of total alkalinity, pH, and acidic metal cations (Fe, Al, and Mn), alongside indices of conservative mixing between river water and seawater (salinity, Na, Mg, K, and Cl), were determined for the Odiel and Tinto branches of the Ria de Huelva estuary between June 10 and 14, 2024 (Figure 1). The fraction of seawater (relative to river water) in each estuarine water sample was calculated using eq 4, using salinity as an index of conservative mixing.

$$F_{\mathrm{sw}}=\frac{S_{\mathrm{sam}}-S_{\mathrm{riv}}}{S_{\mathrm{sw}}-S_{\mathrm{riv}}}$$ 4

Here, *F*_sw_ is the proportion of the estuarine water sample derived from seawater, *S*_sam_ and *S*_sw_ represent the salinity of an estuarine water sample and local seawater, respectively, and *S*_riv_ represents the salinity of the river water endmember sites (Niebla and Gibraleón, Figure 1). The use of salinity measurements in this regard is validated by correlation with other conservative elements (Cl, Ca, Mg, Na, and K) (Supporting Information S.4). An exception is the observation of nonconservative dissolved Ca behavior in the Tinto river branch of the estuary, which is attributed to Ca inputs from an adjacent phosphogypsum waste pile.^37^

The amount of seawater alkalinity consumed per volume of river water discharged through each branch of the estuary (Alk_consumed_) was calculated using eq 5 (see Supporting Information S.5 for derivation).

$${\mathrm{Alk}}_{\mathrm{consumed}}=\frac{F_{\mathrm{sw}-\mathrm{zero}}\times {\mathrm{Alk}}_{\mathrm{sw}}}{1-F_{\mathrm{sw}-\mathrm{zero}}}$$ 5

where Alk_sw_ is the total alkalinity (mM) of the seawater endmember and *F*_sw‑zero_ is the fraction of seawater (eq 2) at the point in each estuarine branch where total alkalinity declines to <1% of Alk_sw_.

The amount of CO_2_ produced per volume of river water discharged through each estuarine branch (mmol CO_2_/L of river water) due to AMD neutralization by carbonate alkalinity was calculated by eq 6:

$$[{\mathrm{CO}}_{2}{]}_{\mathrm{produced}}={\mathrm{Alk}}_{\mathrm{consumed}}\times \frac{\mathrm{Carb}\times {\mathrm{Alk}}_{\mathrm{sw}}}{{\mathrm{Alk}}_{\mathrm{sw}}}$$ 6

where Carb. Alk_sw_ is the sum of HCO_3_^–^ and CO_3_^2–^ concentrations (in mmol/L) within the seawater endmember of the estuary, which was calculated from measurements of pH, total alkalinity, and temperature using the CO2SYS Excel toolbox.^38^ Fluxes of CO_2_ emitted by AMD neutralization in the Ria de Huelva estuary were then calculated by scaling [CO_2_]_produced_ by river discharge (L/time). The key assumption is that the CO_2_ produced via carbonate alkalinity consumption (eq 1) will ultimately be degassed from the estuary/surface coastal waters to the atmosphere. Although exact CO_2_ degassing rates are unknown, this assumption is justified given that these waters will remain in contact with the atmosphere for many years prior to circulation into the deep ocean.^39^

### Carbon Dioxide Emission Estimates Based on Laboratory River Water–Seawater Mixing Experiments

Carbon dioxide emissions per AMD-impacted river water discharged through the Ria de Huelva estuary were also estimated using laboratory neutralization experiments between seawater and river water. The experiments involved titration of a known volume of AMD-impacted river water with seawater to reach an end point of pH 7.5. The seawater alkalinity that was consumed (Alk_consumed_) per volume of neutralized river water during the experiments was calculated using eq 7.

$${\mathrm{Alk}}_{\mathrm{consumed}}=\frac{\left(V_{\mathrm{SW}}\times {\mathrm{Alk}}_{\mathrm{SW}}-V_{\mathrm{final}}\times {\mathrm{Alk}}_{\mathrm{final}}\right)}{V_{\mathrm{RW}}}$$ 7

where *V*_SW_ (L) is the volume of seawater added to the volume of river water (*V*_RW_; L) and *V*_final_ is the total volume of the solution at the end point (*V*_final_ = *V*_SW_ + *V*_RW_). The total alkalinity of the initial seawater is denoted by Alk_SW_ (as defined above), and the total alkalinity of the solution at the end point is denoted by Alk_final_. The CO_2_ produced per volume of AMD-impacted river water was then calculated using eq 6, as described above.

Neutralization experiments were performed with river water from six locations sampled in April and June 2024 (Figure 1). These include samples of Odiel and Tinto endmember river waters collected at Gibraleón and Niebla (samples O1 and T1, respectively) in June 2024 with the aim of replicating the chemistry observed in estuarine water transects collected in that time period. They also include river water samples from these locations collected during the time period of 2–6 April during higher discharge conditions, in addition to four river water samples collected in the upper reaches of the Tinto and Odiel river catchments (Figure 1). These samples span a large range in acidities (eq 2) and compositions of acidic metal cations (Fe, Al, and Mn). Although the latter samples do not represent river waters that are directly discharged through the estuary, their experimental results are used to provide empirical relationships between acidity and Alk_consumed_, which can be used to integrate variability in CO_2_ emission fluxes linked to the hydrochemical dynamics of these river catchments.^24,27^

### Water Sampling Protocols

Samples of river, estuarine, and seawater were collected using a plastic bucket that was prerinsed three times with the sampled water or were sampled directly using polypropylene syringes. River water and seawater endmembers were sampled from locations accessed via the bank or shore. Most of the estuarine transect samples were collected via a boat, although some of the upstream estuarine sites were accessed from locations along the bank and were sampled at different stages of the tidal cycle. Measurements of pH, temperature, oxidation–reduction potential, electrical conductivity, and practical salinity were made in situ using HM Digital COM-300 and HANNA HI98121 (April 2024) as well as HANNA HI98195 (June 2024) water quality probes. All probes were calibrated before use, and single measurements were recorded once the values had stabilized.

Water samples were passed through a 0.22 μm poly­(ether sulfone) capsule filter using polypropylene syringes and collected into high-density polyethene (HDPE) bottles. The syringes and capsule filters were prerinsed with the sampled water prior to collection. Separate bottles of each sample were collected for measurements of cations (Fe, Al, Mn, Ca, Mg, Na, and K), anions (SO_4_^2−^, Cl^−^), total alkalinity, and for river water used for the neutralization experiments. The HDPE bottles used for the cation and experimental aliquots were acid-cleaned using 3 M HCl followed by three rinses with 18.2 MΩ·cm water from a Milli-Q system. The bottles used for anion and alkalinity aliquots were precleaned by rinsing three times with 18.2 MΩ·cm water from a Milli-Q system. The sample aliquots collected for cation concentrations were acidified with trace metal grade 15 M HNO_3_ within 24 h after collection.

### Analytical Techniques

Total alkalinity was determined via Gran titration with either 0.1 or 0.01 M HCl (depending on the sample’s alkalinity), using a Hach digital titrator. Concentrations of the 0.1 and 0.01 M HCl solutions were determined by titration with 1 M NaOH. Cation concentrations (Fe, Al, Mn, Ca, Mg, Na, and K) were determined using microwave plasma atomic emission spectroscopy (MP-AES; 4210 Agilent Technologies). Samples were diluted by factors of 1–250 in a 2% (v/v) solution of distilled HNO_3_, and concentrations were determined using an 11-point calibration line. Accuracy and precision were assessed with repeat measurement of SLRS-6 River Water certified reference material (CRM) (Supporting Information S.7). Concentrations of dissolved Cl^–^ and SO_4_^2–^ were determined by ion chromatography (Dionex ICS6000 from Thermo Fisher Scientific, equipped with an AG17-C guard column, an AS17-C separator column, a KOH eluent generator, and an ADRS 600 2 mm suppressor), with accuracy and precision assessed with repeat measurement of a river water anion CRM, LGC6025. To distinguish between Fe^2+^ and Fe^3+^, unacidified samples (*n* = 18) were analyzed for total Fe and Fe^2+^ using spectrophotometry (Supporting Information S.8). These analyses were conducted at the University of Huelva within 24 h of collection for select samples collected in June 2024, to quantify Fe speciation of the samples at the time of sampling, using a HACH DR/890 colorimeter. The river water samples used for neutralization experiments, collected in both April and June 2024, were subsequently analyzed for Fe^2+^ concentrations several weeks after collection at the University of St Andrews using spectrophotometry (Evolution 220, Thermo Fisher Scientific). While the Fe speciation of the latter measurements may not accurately reflect that of the waters at the time of sampling due to Fe^2+^ oxidation to Fe^3+^, they are taken to reflect the composition of these samples at the time of conducting the neutralization experiments. Full details of the protocols used in the spectrophotometry measurements can be found in the Supporting Information.

## Results and Discussion

### Within-Catchment Silicate Weathering Neutralizes about Half of AMD Acidity in the Odiel and Tinto River Catchments

Dissolved anions in the Odiel and Tinto rivers are dominated by SO_4_^2–^ (93%–97% of negative charge), with the remainder being Cl^–^ (Figure 2). In these catchments, SO_4_^2–^ is derived from sulfide mineral oxidation reactions, while Cl^–^ is mainly derived from atmospheric deposition of sea salt. The negative charge of SO_4_^2–^ therefore provides a measure of the initial acidity in these waters due to pyrite oxidation. At sites just upstream from the Ria de Huelva estuary (Gibraleón and Niebla), anion budgets are charge-balanced by a combination of acidic cations (35–64% of positive charge) and base cations (36%–65% of positive charge) (Figure 2). Correcting for rain inputs of base cations, F_es_ values are 0.37–0.40 and 0.62–0.66 for river water samples collected at Gibraleón (Odiel river) and Niebla (Tinto river), respectively. The river water hydrochemistry data, therefore, imply that in-catchment chemical weathering reactions neutralize ca. 60 and 40% of the initial AMD acidity in the Rio Odiel and Rio Tinto catchments, respectively. The remaining AMD acidity is neutralized upon mixing with seawater in the Ria de Huelva estuary.

**Figure 2:**
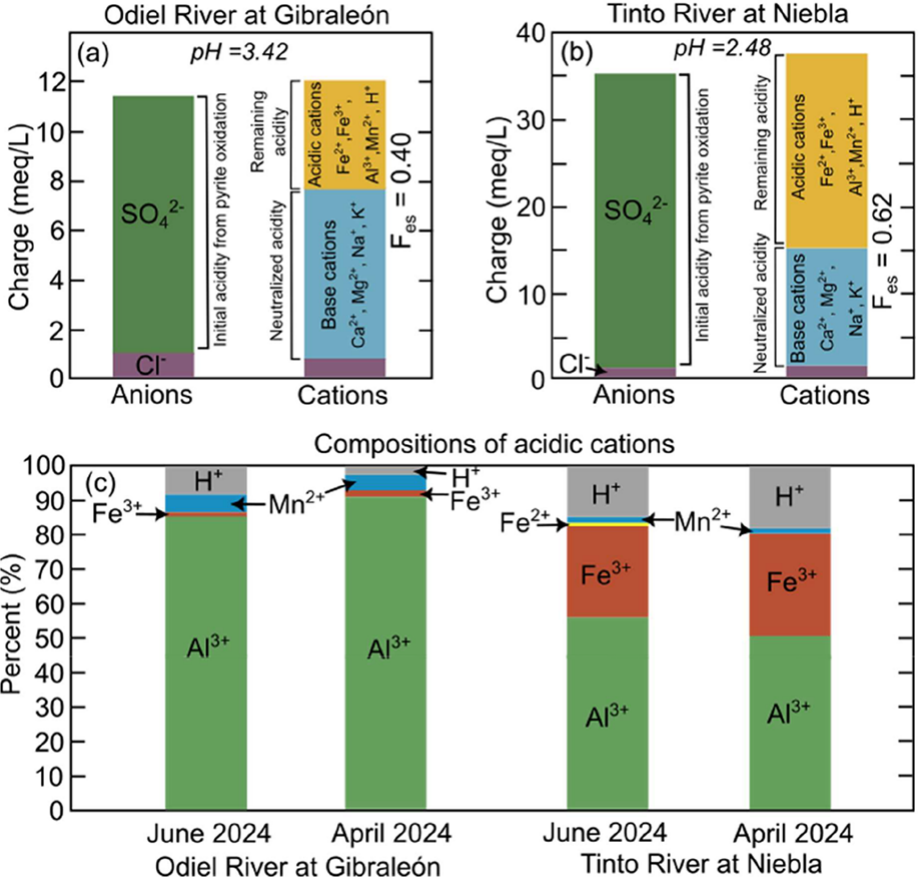
Hydrochemistry of Odiel and Tinto river water samples upstream from the Ria de Huelva estuary. Panels (a) and (b) show anion and cation budgets for the Odiel river at Gibraleón and the Tinto river at Niebla, respectively, sampled in June 2024. Purple fields in the cations column denote base cation charge that is derived from sea salt deposition, constrained using Cl^–^ budgets, while the blue fields denote base cations derived from in-catchment rock weathering. Panel (c) shows the composition of acidic cation-supported positive charge.

The magnitude of CO_2_ emissions resulting from in-catchment AMD neutralization depends on the relative importance of silicate (no CO_2_ emission) vs carbonate (0.5 mol CO_2_ emitted per mol of acidity neutralized) mineral weathering. The composition of base cations in river dissolved loads can be used to distinguish between silicate and carbonate sources.^35^ In general, carbonate weathering typically supplies higher proportions of Ca and Mg relative to Na (Ca/Na ≈ 50, Mg/Na ≈ 10) than silicate weathering (Ca/Na ≈ 0.35, Mg/Na ≈ 0.24)^35^ (Figure 3). These signatures can, however, vary depending on the specific compositions of the local silicate and carbonate lithologies. The geology of the Odiel and Tinto river catchments is dominated by siliciclastic sediments (shales, sandstones, and conglomerates) and mafic to felsic volcanic rocks, which host the VMS deposits.^34,40^ The latter feature higher abundances of minerals enriched in base cations capable of neutralizing AMD.^34,40^ The catchments feature only relatively small areas of carbonate rocks in their lower reaches.^20,41^ The base cation compositions corrected for sea salt inputs of the Odiel and Tinto river dissolved loads feature relatively high Ca/Na (1.6–2.7), which could indicate contributions from carbonate weathering (Figure 3). However, the base cation compositions are enriched in Mg relative to the typical mixing line between silicate and carbonate-derived cation compositions (Mg/Na = 3.9–8.4). These base cation compositions are instead consistent with those for the local volcanic host rocks (rhyolites, andesites, dacites and basalts, and silicate rocks) of VMS deposits of the Iberian Pyrite Belt.^36^ Furthermore, these base cation compositions do not significantly vary between upstream locations directly draining the volcanic-hosted VMS deposits and downstream locations that capture the full range of the catchment’s geology (Figures 1 and 3). Therefore, we interpret the rock-derived base cations within the Odiel and Tinto river dissolved loads to be dominated by weathering of silicate volcanic rocks. This implies that the component of AMD acidity that is neutralized by in-catchment chemical weathering is not associated with CO_2_ emissions.

**Figure 3:**
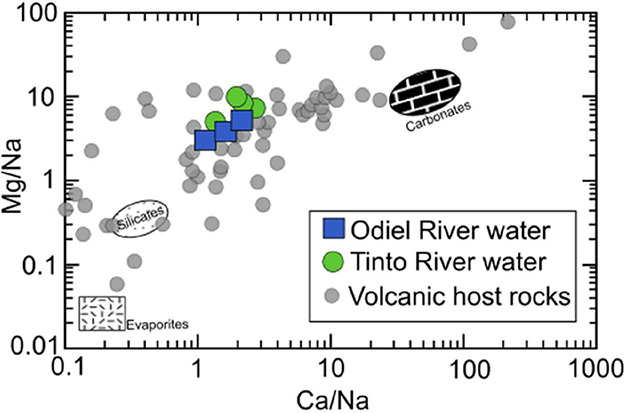
Base cation composition (molar ratios) of Odiel and Tinto river dissolved loads corrected for sea salt inputs relative to the compositions of volcanic host rocks of VMS deposits in the Iberian Pyrite Belt.^36^ Also shown are the base cation compositions typical of being sourced from the weathering of carbonate, silicate, and evaporite rocks.^35^

The remaining acidities of the Odiel and Tinto river dissolved loads that discharge into the Ria de Huelva are 2.37–4.63 and 11.0–22.2 mM, respectively (Figure 2). The variability observed for each river is linked to hydrological conditions, with lower values during the higher discharge conditions sampled in April 2024. The acidity of these dissolved loads is dominated by acidic metals (82–97%) with minor contributions from H^+^ ions (3–18%) (eq 2; Figure 3). For the Odiel river at Gibraleón (pH 3.42–4.20), Al^3+^ dominates acidity (85–91%), with minor contributions from Mn^2+^ (4.5–5.2%), Fe^3+^ (1.2–2.0%), and H^+^ (3–8%). The Tinto river at Niebla, featuring a lower pH (2.48–2.70), has acidity dominated by Al^3+^ (51–56%) and Fe^3+^ (27–30%), with minor contributions from Mn^2+^ (1.5–1.7%) and H^+^ (15–18%).

### Neutralization of AMD in the Ria de Huelva Estuary Drives CO_2_ Emissions of 32 ± 11 kt/yr

Geochemical distributions across the Ria de Huelva estuary trace the near-complete neutralization of river-derived AMD acidity and the coupled consumption of seawater alkalinity (Figure 4). Within both branches of the Ria de Huelva estuary, river-derived dissolved Fe and Al decrease below conservative mixing relationships with increasing salinity (Figure 4). Likewise, total alkalinity decreases below conservative mixing relationships with declining salinity from an initial value of 2.64 mM in the seawater endmember. Total alkalinity and dissolved Fe and Al concentrations converge toward 0 mM at the same point in the estuarine transects, which is marked by a sharp rise in pH. This is consistent with previous studies and reflects the precipitation of Fe and Al oxyhydroxide and oxyhydroxysulfate phases in response to acidity neutralization by seawater alkalinity.^19,20,31,32^ These precipitation reactions release H^+^ ions, buffering the neutralization process, maintaining low pH until the Fe- and Al-hosted acidity has been completely removed. In contrast to dissolved Fe and Al, dissolved Mn displays conservative distributions (only impacted by mixing between river water and seawater endmembers) in the Ria de Huelva estuary (Supporting Information S.2), consistent with the findings of previous studies.^20^ The minor component of AMD acidity supported by Mn^2+^ (Figure 2) therefore plays no role in the consumption of seawater alkalinity in the Ria de Huelva estuary.

**Figure 4:**
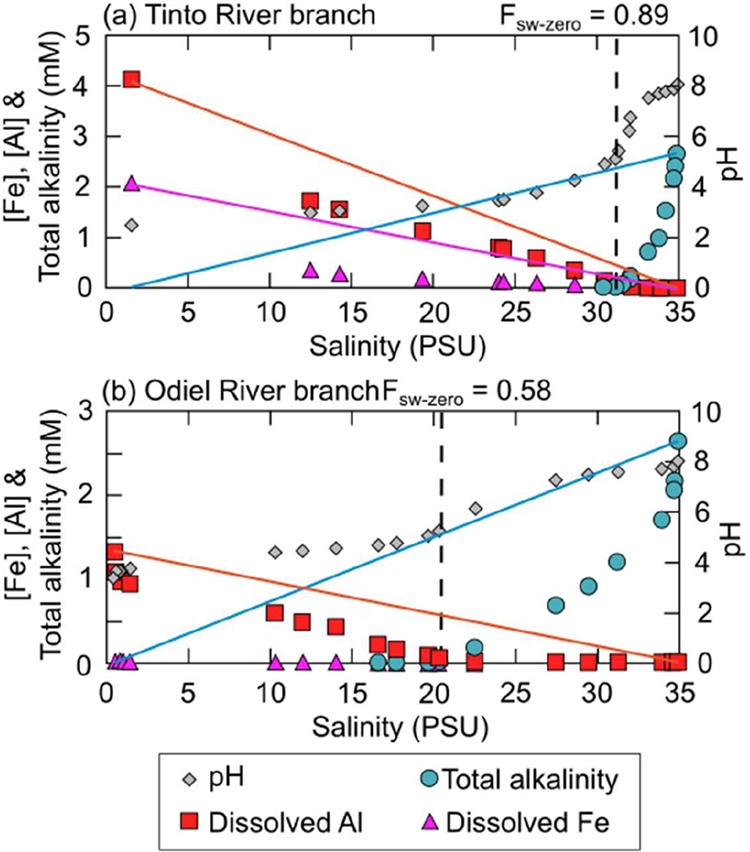
Distributions of geochemical properties across the Tinto river (panel a) and Odiel river (panel b) branches of the Ria de Huelva estuary. Solid lines represent conservative mixing relationships between seawater and river water endmembers for total alkalinity (blue), dissolved Al (red), and dissolved Fe (magenta). Vertical dashed lines denote salinities at which total alkalinity reaches 1% of the initial seawater value. PSU, practical salinity unit.

The salinity at which seawater alkalinity is completely consumed by the neutralization of AMD acidity differs between the two branches of the estuary (Figure 4). Seawater-derived alkalinity is consumed to less than 1% of its initial value at a higher salinity (31.16 g/kg) in the Tinto river branch of the estuary than in the Odiel river branch (20.30 g/kg), corresponding to *F*_sw‑zero_ values of 0.89 and 0.58, respectively (eq 4). These *F*_sw‑zero_ values correspond to Alk_consumed_ values of 20.8 mM and 3.6 mM river water from the Tinto river and Odiel river branches, respectively (eq 5). These field-derived estimates agree within 11% of those quantified by the neutralization experiments (18.7 mM, Tinto river; 3.5 mM, Odiel river). Field and experimental estimates of Alk_consumed_ are positively correlated with river water acidity but are systematically lower by about 20% (Alk_consumed_ = 0.79 × acidity; *R* ^2^ = 0.98, Figure 5). These results show that, on average, only 0.79 mol of seawater alkalinity is consumed for every 1 mol of AMD acidity discharged into the estuary. This acid–base imbalance cannot be explained by the conservative behavior of Mn in the estuary (Supporting Information S.2). Instead, it reflects the role of metal oxyhydroxysulfate mineral precipitation (schwertmannite and basalumnite), resulting in the removal of SO_4_^2–^, and thus reducing the ratio of H^+^ ions produced by these reactions per unit of acidic metal charge loss^7,21^ (Supporting Information S.1). The formation of these minerals, therefore, reduces the stoichiometry between riverine AMD acidity fluxes into the estuary (as defined by eq 2) and resulting CO_2_ emissions due to alkalinity consumption. However, these minerals are metastable and will ultimately convert to metal oxides or oxyhydroxides over time (e.g., goethite, hematite^22^), releasing SO_4_^2–^ and reversing this acid–base imbalance. Over longer time scales, it is therefore expected that seawater alkalinity consumption should have a 1:1 relationship with riverine AMD acidity fluxes into the estuary.

**Figure 5:**
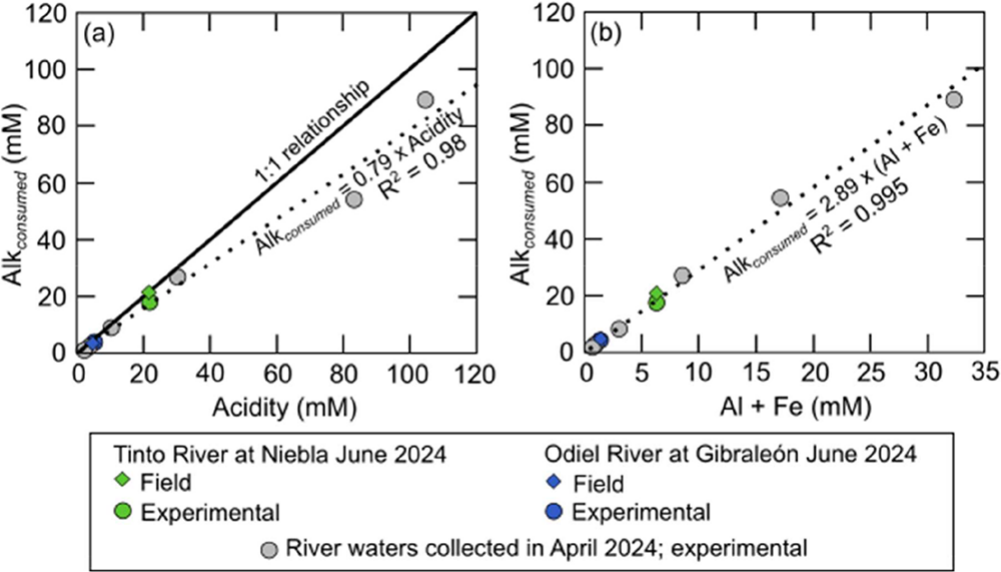
Relationships between Alk_consumed_ and river water acidity (panel a) and river water acidic metal concentrations (panel b). Dashed lines denote linear regressions through the data forced through the y intercept, with the equation of the line and *R* ^2^ values given below. Results for Alk_consumed_ constrained both from the estuarine transects (field) and from neutralization experiments (experimental) are shown for samples collected in June 2024. For river water samples collected in April 2024, including those from locations in the upper reaches of the catchments, Alk_consumed_ are only available from neutralization experiments.

Based on the temperature (24.71 °C), pH (8.03), and total alkalinity (2.64 mM) of the local seawater endmember of the Ria de Huelva estuary sampled in June 2024, 0.87 mol of CO_2_ is produced per mole of Alk_consumed_.^38^ Coupled with the field and experiment constraints on Alk_consumed_, this yields 1.6 and 3.1 (Odiel), and 7.1 and 18.2 (Tinto) mol of CO_2_ produced per L of river water discharged into the Ria de Huelva for the April and June 2024 samples (Supporting Information S.3). Scaling these numbers by the river discharge at the times of sampling yields total CO_2_ emission fluxes from the Ria de Huelva estuary of 171 and 16 ktCO_2_/yr for the April and June 2024 hydrological conditions, respectively. This variability in CO_2_ emission flux estimates reflects seasonal differences in both river discharge and river water AMD acidity concentrations, which are known to covary in these river catchments.^24,25^ The long-term (seasonally averaged) CO_2_ emission flux associated with AMD neutralization in these catchments, relevant to quantifying the importance of this CO_2_ emission source to metal production carbon footprints, is likely to be within this range.

Previous studies have used time series data to constrain annually averaged fluxes of dissolved Fe and Al into the Ria de Huelva estuary ranging between 0.6 and 13 kt/yr.^24–29^ Data from our field survey and neutralization experiments show that 2.89 mol of seawater alkalinity is consumed for every mole of Fe and Al discharged into the estuary (Figure 5b). This empirical relationship includes the consumption of alkalinity by acidity supported by these metals in addition to that supported by H^+^ ions, noting that concentrations of these species tend to covary in these rivers.^42^ It also accounts for the acid–base imbalance (0.79 mol of alkalinity consumed per 1 mol of acidity) due to sulfate removal by metal oxyhydroxysulfate precipitation (Figure 5a). Coupling this relationship to literature constraints for Fe and Al fluxes into the Ria de Huelva estuary for the years 2001/2002, 2002/2003, and 2005/2006 yields a CO_2_ emission flux of 32 ± 11 ktCO_2_/yr (mean ± 2SD) (Supporting Information S.3). These three years feature both Fe and Al flux estimates for both the Tinto and Odiel rivers, allowing calculation of the full CO_2_ flux from the estuary.^24,27^ This flux estimate integrates seasonal variability in Odiel and Tinto river AMD fluxes and reassuringly lies within the range of our constraints for the two endmember hydrological conditions sampled in April and June 2024.

### The Importance of AMD-Associated CO_2_ Emissions to Metal Production Carbon Footprints

Copper is the main metal that has been historically produced from the Iberian Pyrite Belt.^11^ Accurate estimates of the total mass of copper produced to date are lacking; however, it is estimated that 245 Mt of massive sulfide ore was extracted from this region between 1873 and 2001.^14^ Assuming average Cu ore grades of 0.4–5 wt % ^11,13^ yields an estimate of 980–12250 kt of Cu produced. This equates to an average Cu production rate of 8–96 kt Cu per year during this time period. While subject to uncertainty, these estimates are reasonable given that the largest mine in this region, the Rio Tinto mine, alone produced ca. 5–25 kt Cu/yr during this period.^43^ Normalizing our estimate of CO_2_ emissions via AMD neutralization of 32 ± 11 ktCO_2_/yr to these estimates of Cu production rates yields between 0.3 and 4.2 t CO_2_ per t Cu (Figure 6). These estimates overlap within the same range as conventional carbon footprints for Cu production of 1–9 t CO_2_ per t Cu.^44,45^

**Figure 6:**
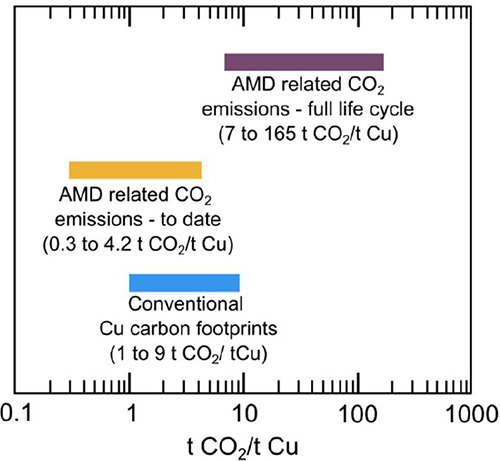
Comparisons of estimates of AMD-related CO_2_ emissions per t Cu production to conventional Cu production carbon footprints.^44,45^

The comparison above highlights the importance of CO_2_ emissions arising from AMD neutralization in the metal production carbon footprints. However, it suffers from uncertainty due to AMD production and neutralization being temporally disconnected from associated metal production. Waste sulfide minerals from the mining process can take decades to millennia to fully oxidize.^8^ The CO_2_ emissions arising from AMD neutralization associated with the historic Cu production from this region will therefore continue to accumulate over this period of sulfide mineral oxidation. This means that our comparison above underestimates the full life cycle of AMD-related CO_2_ emissions per unit of Cu production. The potential AMD-related CO_2_ emissions per unit metal production, integrated over the time period it takes to fully oxidize the associated waste sulfides, will be dependent on two key factors. First is the ratio of metal of economic interest to waste sulfide minerals in the excavated material. This determines the amount of acidity that will ultimately be generated per unit of metal production. Second is the pathways by which this acidity will be neutralized in the downstream environment (silicate vs carbonate weathering, and reaction with seawater alkalinity), which will determine the CO_2_ emissions per unit of acidity.

The oxidative weathering of pyrite (FeS_2_), the dominant sulfide mineral in VMS deposits of the Iberian Pyrite Belt, produces 4 mol of acidity per mol of pyrite (Supporting Information S.1). Assuming an ore composition of 50–100 wt % pyrite with 0.4–5 wt % Cu^11,13^ results in a potential acidity production of 3.3 × 10^5^ moles to 8.3 × 10^6^ moles per t Cu. Results from our study show that approximately half of the AMD acidity produced in this region is neutralized by in-catchment chemical weathering, associated with no CO_2_ emission. The remaining AMD acidity is neutralized by seawater alkalinity during estuarine mixing with ∼0.9 mol of CO_2_ emitted per mol of acidity. Application of these constraints yields potential AMD-related CO_2_ emissions per unit of Cu production in the Iberian Pyrite belt of 7–165 t CO_2_ per t Cu, over the full oxidation time scale of the extracted sulfides. This estimate exceeds those of conventional Cu production carbon footprints of 1–9 t CO_2_ per t Cu by more than an order of magnitude^44,45^ (Figure 6). The full oxidative weathering of the 245 Mt of massive sulfides historically excavated in this region will result in a total CO_2_ emission of 78–156 MtCO_2_. However, at the current rate of CO_2_ emission (32 ± 11 ktCO_2_/yr), it would take ca. 2000–7000 years to release this full CO_2_ emission budget, highlighting the longevity of this CO_2_ emission source.

The findings of this study show that CO_2_ emissions related to AMD neutralization can dominate the carbon footprints of metal production from sulfide-rich ore deposits over the full life cycle of the associated waste sulfide minerals. This warrants the consideration of this CO_2_ emission source in metal production life-cycle assessments. The magnitude of this CO_2_ emission source, relative to metal production, will depend on the ratio of sulfide minerals to metal abundance in the ore material and the neutralization pathways of the resulting AMD in the downstream environment. The high sulfide mineral abundances of the VMS deposits in the Iberian Pyrite Belt likely make AMD-related CO_2_ emissions from this mining region among the highest in the world. Other deposit types, with lower sulfide mineral-to-metal ratios, are predicted to be associated with lower magnitude AMD-related CO_2_ emissions. The use of AMD remediation approaches may help mitigate associated CO_2_ emissions by impacting AMD generation rates or neutralization pathways. Source control techniques focusing on managing the storage of waste sulfide minerals could slow the rate of AMD-associated CO_2_ emissions. However, migration control techniques that treat AMD-impacted waters often involve neutralization using carbonate minerals, resulting in CO_2_ emissions.^46^ The extent to which remediation approaches impact AMD-related CO_2_ emissions requires careful consideration in future mining developments. The approach outlined in this study to quantify AMD-related CO_2_ emissions as a function of metal-to-sulfide mineral ratios and neutralization pathways can be applied in other mining areas to address these questions.

## Supplementary Material



## Data Availability

The data underlying this study are openly available in the HydroShare repository at http://www.hydroshare.org/resource/91a4af803f9a49a383f91fd551f7aa0e.

## Notes

Extended details on the chemistry of acid mine drainage; demonstration of the near-conservative behavior of dissolved Mn in the Ria de Huelva estuary; details on the calculation of CO_2_ emissions associated with acid mine drainage neutralization in the Ria de Huelva estuary; mixing relationships for a range of conservative indices of river water–seawater mixing in the Ria de Huelva estuary; full derivation of the mass balance equation used to quantify Alk_consumed_ from estuarine field data (eq 5); extended details on the acid mine drainage neutralization experiments; data quality details for microwave plasma atomic emission spectroscopy and ion chromatography analyses; and protocols for spectrophotometry measurements of Fe speciation (PDF)

## References

[1] Azadi M., Northey S. A., Ali S. H., Edraki M. (2020). Transparency on greenhouse
gas emissions from mining to enable climate change mitigation. Nat. Geosci..

[2] Atekwana E. A., Fonyuy E. W. (2009). Dissolved inorganic
carbon concentrations and stable
carbon isotope ratios in streams polluted by variable amounts of acid
mine drainage. Journal of Hydrology.

[3] Raymond P. A., Oh N. H. (2009). Long-term changes
of chemical weathering products in rivers heavily
impacted from acid mine drainage: Insights on the impact of coal mining
on regional and global carbon and sulfur budgets. Earth and Planetary Science Letters.

[4] Nordstrom D. K. (2011). Mine waters:
Acidic to circmneutral. Elements.

[5] Simate G. S., Ndlovu S. (2014). Acid mine drainage:
Challenges and opportunities. Journal of Environmental
Chemical Engineering.

[6] Nordstrom D. K. (2000). Advances
in the hydrogeochemistry and microbiology of acid mine waters. International Geology Review.

[7] Bigham J. M., Nordstrom D. K. (2000). Iron and
aluminum hydroxysulfates from acid sulfate
waters. Reviews in mineralogy and geochemistry.

[8] Younger P. L. (1997). The longevity
of mine water pollution: a basis for decision-making. Sci. Total Environ..

[9] Brown A. M., Bass A. M., Garnett M. H., Skiba U. M., Macdonald J. M., Pickard A. E. (2024). Sources and controls of greenhouse
gases and heavy
metals in mine water: A continuing climate legacy. Sci. Total Environ..

[10] Leistel J. M., Marcoux E., Thiéblemont D., Quesada C., Sánchez A., Almodóvar G. R., Pascual E., Saez R. J. M. D. (1997). The volcanic-hosted
massive sulphide deposits of the Iberian Pyrite Belt Review and preface
to the Thematic Issue: Review and preface to the Thematic Issue. Miner. Deposita.

[11] Tornos F. (2006). Environment
of formation and styles of volcanogenic massive sulfides: The Iberian
Pyrite Belt. Ore Geology Reviews.

[12] Olias M., Nieto J. M. (2015). Background conditions
and mining pollution throughout
history in the Rio Tinto (SW Spain). Environments.

[13] Strauss, G. K. ; Madel, J. ; Fdez Alonso, F. Exploration practice for strata-bound volcanogenic sulphide deposits in the Spanish-Portuguese Pyrite Belt: Geology, geophysics, and geochemistry. In Time-and strata-bound ore deposits; Springer Berlin Heidelberg: Berlin, Heidelberg, 1977; pp 55–93.

[14] Moreno Bolaňos, F. Mineral extraido en minas de Riotino (1725–2002). In Rio Tinto. Historia, Patrimonio Minero y Turismo Cultural, Pérez Macias, J. A. ; Dalgado Dominguez, A. ; Pérez López, J. M. ; Garcia Delgado, F. J. , Eds.; Servicio de Publicaciones: Universidad de Huelva, 2011; pp 761–770.

[15] Elbaz-Poulichet F., Morley N. H., Beckers J. M., Nomerange P. (2001). Metal fluxes
through the Strait of Gibraltar: the influence of the Tinto and Odiel
rivers (SW Spain). Marine Chemistry.

[16] Romero-Matos J., Sanchez-Lopez L., Leon R., Runkel R. L., Nordstrom D. K., Canovas C. R., Macias F., Nieto J. M. (2026). Characterization
and modelling approach for planning restoration strategies in a complex
basin affected by acid mine drainage. Journal
of Environmental Management.

[17] Kirby C. S., Cravotta C. A. (2005). Net alkalinity and net acidity 1:
Theoretical considerations. Appl. Geochem..

[18] Kirby C. S., Cravotta C. A. (2005). Net alkalinity and
net acidity 2: Practical considerations. Appl.
Geochem..

[19] Pérez-López R., Millán-Becerro R., Dolores-Basallote M., Carrero S., Parviainen A., Freydier R., Macias F., Cánovas C. R. (2023). Effects
of estuarine water mixing on the mobility of
trace elements in acid mine drainage leachates. Mar. Pollut. Bull..

[20] Papaslioti E.-M., Giampouras M., Sánchez-López L., Basallote M.-D., Freydier R., Cánovas C. R., Pérez-López R. (2024). Temporal dynamics
of contaminants
in an estuarine system affected by acid mine drainage discharges. Sci. Total Environ..

[21] Bigham J. M., Schwertmann U., Traina S. J., Winland R. L., Wolf M. (1996). Schwertmannite
and the chemical modelling of iron in acid sulfate waters. Geochim. Cosmochim. Acta.

[22] Tosca N. J., McLennan S. M., Dyar M. D., Sklute E. C., Michel F. M. (2008). Fe oxidation
processes at Meridiani Planum and implications for secondary Fe mineralogy
on Mars. J. Geophys. Res..

[23] Hilton R. G., West A. J. (2020). Mountains, erosion and the carbon cycle. Nature Reviews Earth & Environment.

[24] Olías M., Cánovas C. R., Nieto J. M., Sarmiento A. M. (2006). Evaluation
of the dissolved contaminant load transported by the Tinto and Odiel
rivers (South West Spain). Appl. Geochem..

[25] Cánovas C. R., Hubbard C. G., Olías M., Nieto J. M., Black S., Coleman M. L. (2008). Hydrochemical variations
and contaminant load in the
Río Tinto (Spain) during flood events. Journal of Hydrology.

[26] Galván L., Olías M., Cerón J. C., Cánovas C. R., Pérez-López R., Nieto J. M. (2013). Assessment
of the
dissolved pollutant flux of the Odiel River (SW Spain) during a wet
period. Sci. Total Environ..

[27] Nieto J. M., Sarmiento A. M., Canovas C. R., Olias M., Ayora C. (2013). Acid mine
drainage in the Iberian Pyrite Belt: 1. Hydrochemical characteristics
and pollutant load of the Tinto and Odiel rivers. Environmental Science Pollution Research.

[28] Galván L., Olías M., Cánovas C. R., Sarmiento A. M., Nieto J. M. (2016). Hydrological modelling
of a watershed affected by acid
mine drainage (Odiel River, SW Spain). Assessment of the pollutant
contributing areas. Journal of Hydrology.

[29] Olías M., Cánovas C. R., Macías F., Basallote M. D., Nieto J. M. (2020). The evolution of
pollutant concentrations in a river
severely affected by acid mine drainage: Río Tinto (SW Spain). Minerals.

[30] Olías, M. ; Cánovas, C. R. ; Basallote, M. D. ; Macías, F. ; Nieto, J. M. ; Pérez-López, R. ; Sarmiento, A. M. The Problem of Acid Mine Drainage in the Iberian Pyrite Belt (IPB): Diagnosis and Treatment Measure; Servicio de Publicaciones de la Universidad de Huelva, 2024. https://www.uhu.es/publicaciones/?q=libros&code=1347.

[31] Hierro A., Olias M., Ketterer M. E., Vaca F., Borrego J., Cánovas C. R., Bolivar J. P. (2014). Geochemical behavior of metals and
metalloids in an estuary affected by acid mine drainage (AMD). Environmental Science Pollution Research.

[32] Asta M. P., Calleja M. L., Pérez-López R., Auqué L. F. (2015). Major hydrogeochemical
processes in an acid mine drainage affected estuary. Mar. Pollut. Bull..

[33] Lehner B., Grill G. (2013). Global river hydrography
and network routing: baseline data and new
approaches to study the world’s large river systems. Hydrological Processes.

[34] Oliveira, J. T. ; Quesada, C. ; Pereira, Z. ; Matos, J. X. South Portuguese Terrane. In The Geology of Iberia: A Geodynamic Approach, Vol. 2: The Variscan Cycle, Quesada, C. ; Oliveira, J. T. , Eds., 2019; pp 173–206.

[35] Gaillardet J., Dupré B., Louvat P., Allègre C. J. (1999). Global
silicate weathering and CO_2_ consumption rates deduced from
the chemistry of large rivers. Chem. Geol..

[36] Mitjavila J., Martí J., Soriano C. (1997). Magmatic Evolution and Tectonic Setting
of the Iberian Pyrite Belt Volcanism. Journal
of Petrology.

[37] Millán-Becerro R., Pérez-López R., Cánovas C. R., Macias F., León R. (2023). Phosphogypsum weathering and implications
for pollutant discharge into an estuary. Journal
of Hydrology.

[38] Pierrot, D. ; Epitalon, J.-M. ; Orr, J. C. ; Lewis, E. ; Wallace, D. W. R. , MS Excel program developed for CO2 system calculations – version 3.0, 2021, GitHub repository, https://github.com/dpierrot/co2sys_xl.

[39] Millot, C. ; Taupier-Letage, I. Circulation in the Mediterranean Sea. In The Mediterranean Sea. Handbook of Environmental Chemistry, Saliot, A. , Ed.; Springer: Berlin, Heidelberg, 2005; Vol. 5K.

[40] Tornos, F. ; Barriga, F. J. A. S. ; Marcoux, E. ; Pascual, E. ; Pons, J. M. ; Relvas, J. M. R. S. ; Velasco, F. ; Large, R. ; Blundell, D. The Iberian pyrite belt. Database on Global VMS Districts, Codes-Geode, 2000; pp 19–52.

[41] Cánovas C. R., Olías M., Cerón J. C., Nieto J. M. (2005). Caracterización
hidroquímica de los arroyos que vierten a la Ría de
Huelva. Geogaceta.

[42] Olías M., Nieto J. M., Sarmiento A. M., Cerón J. M., Cánovas C. R. (2004). Seasonal water quality variations
in a river affected
by acid mine drainage: The Odiel River (South West Spain). Sci. Total Environ..

[43] Harvey C. E. (1981). The Rio
Tinto Company: An economic history of a leading international mining
concern, 1873–1954. J. Econ. Hist..

[44] Kuckshinrichs W., Zapp P., Poganietz W. R. (2007). CO_2_ emissions of global
metal-industries: The case of copper. Applied
Energy.

[45] Northey S., Haque N., Mudd G. (2013). Using sustainability reporting to
assess the environmental footprint of copper mining. Journal of Cleaner Production.

[46] Martinez N. M., Basallote M. D., Meyer A., Cánovas C. R., Macias F., Schneider P. (2019). Life cycle
assessment of a passive
remediation system for acid mine drainage: Towards more sustainable
mining activity. Journal of Cleaner Production.

