# Supplementary material for: Carbon Dioxide Emissions from Acid Mine Drainage Neutralization Are a Major Component of Metal Mining Carbon Footprints
Source: Environ Sci Technol. 2026 Jul 22;60(30):20990–8. doi: 10.1021/acs.est.6c05684 (PMC13449882; doi:10.1021/acs.est.6c05684)
Supplement: Supplementary file 1 [file es6c05684_si_001.pdf]

Supporting Information for

Carbon dioxide emissions from acid mine drainage neutralization are a major component of metal mining carbon footprints

Stanisław Kołodziejski[1], Eva E. Stüeken[1], Laura Sánchez López[2], Jose Miguel Nieto[2], Carlos R. Cánovas[2], Rafael Pérez-López[2], Luke Bridgestock[1]

[1]School of Earth and Environmental Sciences, University of St Andrews, St Andrews, KY16 9TS, UK

[2]Department of Earth Sciences & Research Center on Natural Resources, Health and the Environment, University of Huelva, Huelva, 21071, Spain

Pages = 16, Figures = 6, Tables =5

S.1. *Acid mine drainage chemistry*

Acid mine drainage is produced by the oxidative weathering of sulfide minerals, principally pyrite (Simate and Ndlovu, 2014; eqn. S1).

$$FeS_2 + 3.5O_2 + H_2O \rightarrow Fe^{2+} + 2H^+ + 2SO_4^{2-} \quad \text{(eqn. S1)}$$

These reactions release both H^+ ions and metal cations into solution, which are charge-balanced by sulfate ions (SO_4^{2-}). The acidity of acid mine drainage (AMD) is supported by both free H^+ (as measured by pH) and acidity-producing metals, principally Fe^{2+}, Fe^{3+}, Al^{3+} and Mn^{2+} (Kirby and Cravotta, 2005ab). Increases in pH due to neutralization reactions (eqn. 1; main article) promote the precipitation of these metal species as oxyhyrdoxide minerals, which in turn releases further H^+ ions equivalent to the charge of the metal species (eqn. S2 and S3).

$$Fe^{3+} + 3H_2O \rightarrow Fe(OH)_3 + 3H^+ (ferrihydrate) \quad \text{(eqn. S2)}$$

$$Al^{3+} + 3H_2O \rightarrow Al(OH)_3 + 3H^+ (gibbsite) \quad \text{(eqn. S3)}$$

These reactions buffer the rise in pH upon AMD neutralization until these acidity-producing metals have quantitatively precipitated from solution (Pérez-López et al., 2023). The acidity of AMD can, therefore, be defined as the sum of the positive charge of both H^+ ions and acidic metal species held in solution (Kirby and Cravotta, 2005ab) (eqn. 2, main article). Based on this definition, oxidative weathering of pyrite (FeS_2) produces 4 moles of acidity ($2H^+$ and Fe^{2+}) per mole of pyrite (eqn. S1).

While equation 2 (main article) provides a good approximation of AMD acidity, it can be impacted by uncertainty in the protonation of SO_4^{2-} species and the extent of Fe hydrolysis in solution at a given pH (Kirby and Cravotta, 2005a). Further uncertainty in this approximation

of AMD acidity stems from the fact that acidic metals tend to precipitate as oxyhydroxysulfates (e.g., schwertmannite, eqn. S4, and basaluminite, eqn. S5), rather than oxyhydroxides (eqn. S2 and S3) at low pH (<4.5; Bigham et al., 1996).

$$8Fe^{3+} + 12.5H_2O + 1.75SO_4^{2-} \rightarrow Fe_8O_8(OH)_{4.5}(SO_4)_{1.75} + 20.5H^+ \quad \text{(eqn. S4)}$$

$$4Al^{3+} + 10H_2O + SO_4^{2-} \rightarrow Al_4(OH)_{10}SO_4 + 10H^+ \quad \text{(eqn. S5)}$$

Incorporation of SO_4^{2-} in place of OH^- into these minerals reduces the stoichiometry of acidic metal charge to protons assumed in eqn. 2 (main article). For example, during schwertmannite precipitation, $2.6H^+$ ions are produced per Fe^{3+} atom removed from solution, as opposed to $3H^+$ ions per Fe^{3+} atom removed assumed in eqn. 2. For basaluminite precipitation, only $2.5H^+$ ions are produced per Al^{3+} atom removed from solution, as opposed to $3H^+$ ions per Al^{3+} atom removed assumed in eqn. 2. This means that eqn. 2 will overestimate the proton acidity produced by about 80% if Fe^{3+} and Al^{3+} removal is dominated by oxyhydroxysulfate (eqn. S4 and S5) rather than oxyhydroxide precipitation eqn. S2 and S3) These metal oxyhydroxysulfates however, are metastable and will ultimately convert oxyhydroxides and oxides, reversing this proton-metal cation balance (Bigham et al., 1996, Tosca et al., 2008).

S.2. *Near conservative behaviour of dissolved Mn in the Ria de Huelva estuary*

Unlike dissolved Fe and Al, dissolved Mn features conservative distributions within the Ria de Huelva estuary that can be solely explained by river water – seawater mixing with no removal by precipitation reactions (Supplementary Fig. 1). This implies that Mn^{2+} does not play a role in consuming seawater alkalinity in the estuary. However, given the relatively minor contribution of Mn^{2+} to river water acidity (Fig. 2, main article), not including Mn^{2+} in

calculations of river water acidity (eqn. 2, main article) makes only a very minor difference in the relationship between $Alk_{consumed}$ and acidity (Supplementary Fig. 2).

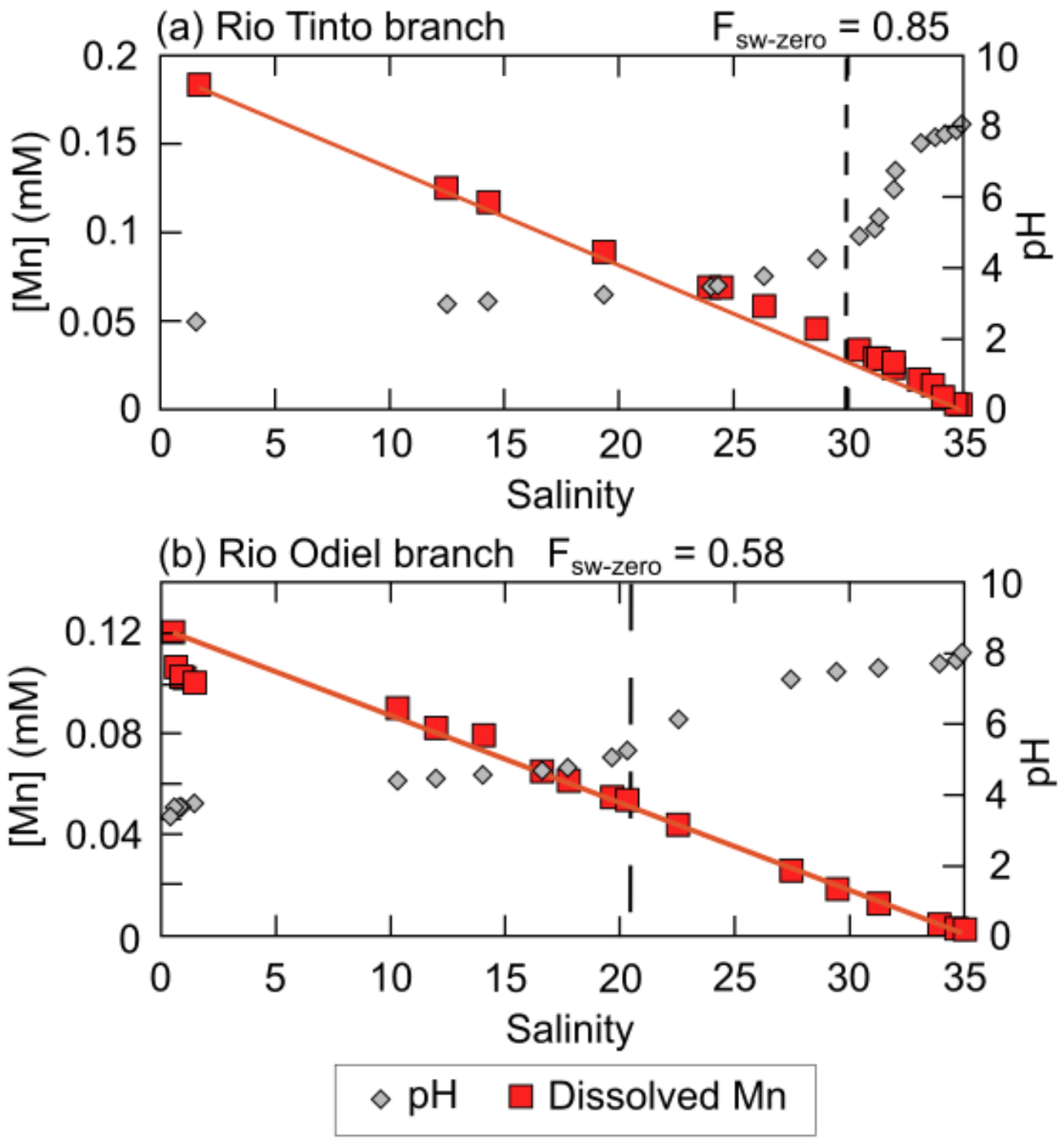

Supplementary Figure 1, Distributions of dissolved Mn in the Ria de Huelva estuary as a function of salinity. Solid red lines represent conservative mixing relationships between seawater and river water endmembers. Vertical dashed black lines denote $F_{sw\text{-}zero}$, the fraction of seawater in the estuarine mixture (F_{sw}) at which total alkalinity reaches 0 (eqn. 4 and 6).

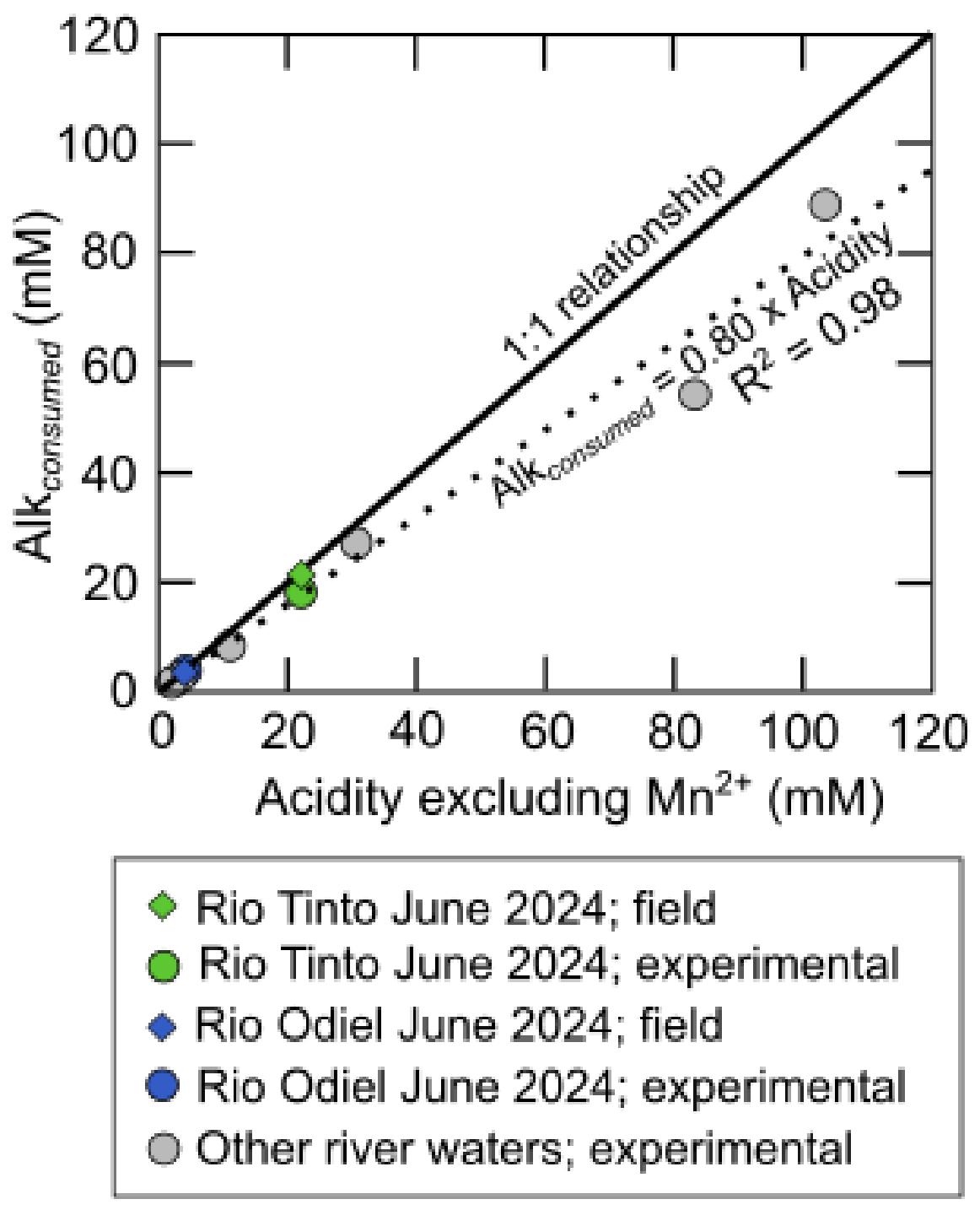

Supplementary Figure 2, Relationship between Alk$_{consumed}$ and river water acidity excluding Mn^{2+}.

S.3 *Results of CO_2 emissions calculations for AMD neutralization in the Ria de Huelva estuary*

Supplementary Table 1, Carbon dioxide emission estimates associated with AMD neutralization in the Ria de Huelva estuary

Estuary branch	Time period	Alk consumed (mM)	CO_2 produced (mM)	Discharge (m^3/s) [a]	CO_2 emission ($ktCO_2/yr$)
Rio Odiel	April 2024	1.9	1.6	11.7	27
	June 2024	3.6	3.1	3.65	16
Rio Tinto	April 2024	8.4	7.1	14.6	144
	June 2024	20.8	18.2	0	0
Total	April 2024				171
	June 2024				16

[a] Discharge data obtained from the Consejeria de Agricultura, Pesca, Agua y Desarrollo Rural, Dirección General de Infraestructuras del Agua; https://www.redhidrosurmedioambiente.es/saih/datos/a/la/carta

While the gage on the lower section of the Rio Tinto recorded 0 m^3/s during sampling during June 2024, visual inspection of the river revealed a small flow, likely ~0.001m^3/s.

Supplementary Table 2, Annually averaged estimates of CO_2 emissions associated with AMD neutralization in the Ria de Huelva estuary

Year	Fe (t/yr)			Al (t/yr)			CO_2 emission[c]
	Rio Odiel	Rio Tinto	Total	Rio Odiel	Rio Tinto	Total	($ktCO_2$/yr)
2001/2002[a]	1376	2518	3894	4439	957	5396	30
2002/2003[a]	2910	3518	6428	4675	1492	6167	38
2005/2006[b]	875	2504	3379	4364	874	5238	28

[a] Annually averaged Fe and Al flux estimates from Olias et al. (2006)

[b] Annually averaged Fe and Al flux estimates from Nieto et al. (2013)

[c] Calculated by multiplying the moles of Fe + Al discharged into the estuary by both rivers by factors of 2.89 (to scale to $Alk_{consumed}$; Fig. 5b, main article) and 0.87 (R_{carb}: Molar ratio of C stored as HCO_3^{2-} and CO_3^{2-} to the total alkalinity of local seawater).

S.4. *Conservative indices of river water – seawater mixing in the Ria de Huelva estuary*

Strong (R^2 > 0.99) linear relationships exist between salinity and other potential indices of conservative mixing (Ca, Mg, Na, K, Cl) in the Ria de Huelva estuary (Supplementary Fig. 3). This is consistent with relationships produced by two endmember (river water – seawater) conservative mixing. An exception to this is dissolved Ca in the Rio Tinto branch of the estuary where data deviate to higher values than those predicted by conservative mixing between river water and seawater, resulting in a lower R^2 of 0.903. This is interpreted to reflect the addition of dissolved Ca from a third endmember, likely the dissolution of a phosphogypsum waste pile that is situated on the banks of this estuarine branch (Millán-Becerro et al., 2023).

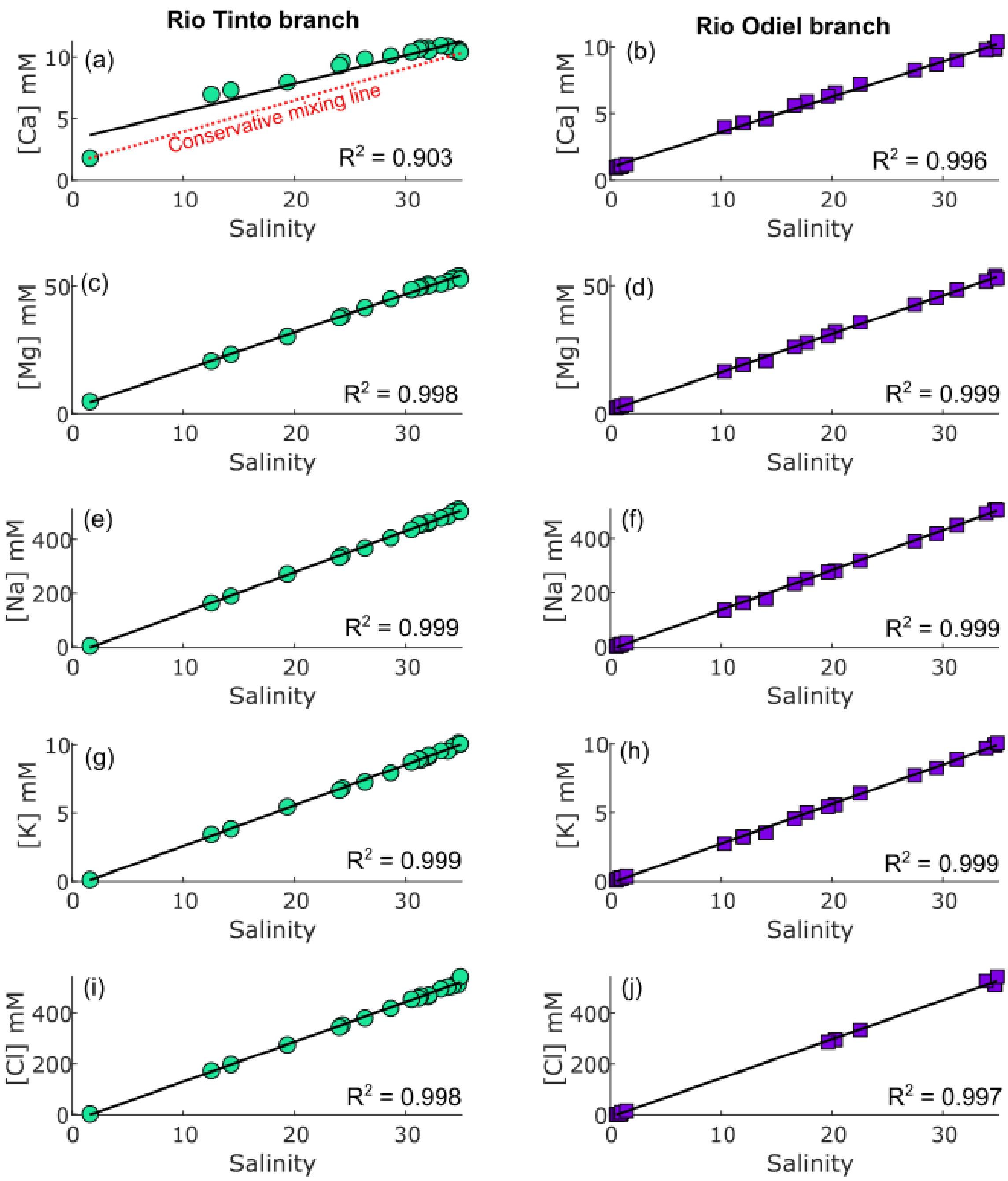

Supplementary Fig. 3, Relationships between salinity and other potential indices of conservative mixing within the Ria de Huelva estuary. Panels (a), (c), (e), (g) and (i) show data for the Rio Tinto branch of the estuary, while panels (b), (d), (f), (h) and (j) show data for the Rio Odiel branch of the estuary. Solid black lines are linear regressions. In panel (a) the dashed red line shows the expected conservative mixing relationship between the river and seawater endmembers.

S.5. *Derivation of equation to constrain $Alk_{consumed}$ (eqn. 5; main article)*

The total alkalinity of an estuarine water sample (Alk_{es}) depends on the proportion of river water and seawater in the mixture (F_{sw}), the total alkalinity of these 2 endmembers (Alk_{riv} and Alk_{sw}) and the amount of alkalinity that has been consumed by AMD neutralization reactions:

$$Alk_{es} = Alk_{riv}(1-F_{sw}) + Alk_{sw}F_{sw} - Alk_{consumed*} \qquad \text{(eqn. S6)}$$

Where Alk_{es}, Alk_{riv} and Alk_{sw} are the total alkalinities of the estuarine mixture, river water and seawater endmembers respectively in mM, F_{sw} is the fraction of seawater in the estuarine mixture (eqn. 4, main article), and $Alk_{consumed*}$ is the amount of total alkalinity that has been consumed in mmole per L of estuarine water (the sum of both river and seawater components). Note that the latter differs from $Alk_{consumed}$ defined in the main article, which reflects the mmoles of total alkalinity consumed per L of river water in the estuarine mixture.

In the Ria de Huelva, Alk_{riv} = 0 mM. Furthermore, at $F_{sw\text{-}zero}$ (fraction of seawater in the estuary mixture at the point where total alkalinity tends towards 0 mM), $Alk_{es} \approx$ 0mM leading to the simplification:

$$0 = Alk_{sw}F_{sw\text{-}zero} - Alk_{consumed*} \qquad \text{(eqn. S7)}$$

Rearranging for $Alk_{consumed*}$:

$$Alk_{consumed*} = Alk_{sw}\, F_{sw\text{-}zero} \qquad \text{(eqn. S8)}$$

Finally, to covert from $Alk_{consumed*}$ (mmole per L estuarine water) to $Alk_{consumed}$ (mmole per L river water in estuarine mixture), divide by the fraction of the estuarine water that is river water (1- $F_{sw\text{-}zero}$) to arrive at eqn. 5 (main article):

$$Alk_{consumed} = \frac{F_{sw-zero} \times Alk_{sw}}{1 - F_{sw-zero}} \qquad \text{(eqn. 5, main article)}$$

S.6. Neutralization experiments

The experimental setup consisted of a borosilicate glass beaker containing AMD impacted water, a Hanna Instruments HI98195 multiparameter meter to measure pH, ORP, electrical conductivity and temperature during the experiments, and a burette to supply seawater (Supplementary Fig. 4). Experiments were mixed with a magnetic stir bar and were conducted at room temperature (20˚C). Seawater was added in increments, before allowing the solution to equilibrate for between 5 minutes and 60 minutes, based on initial tests on the timescales needed for pH to converge to within 0.1 units.

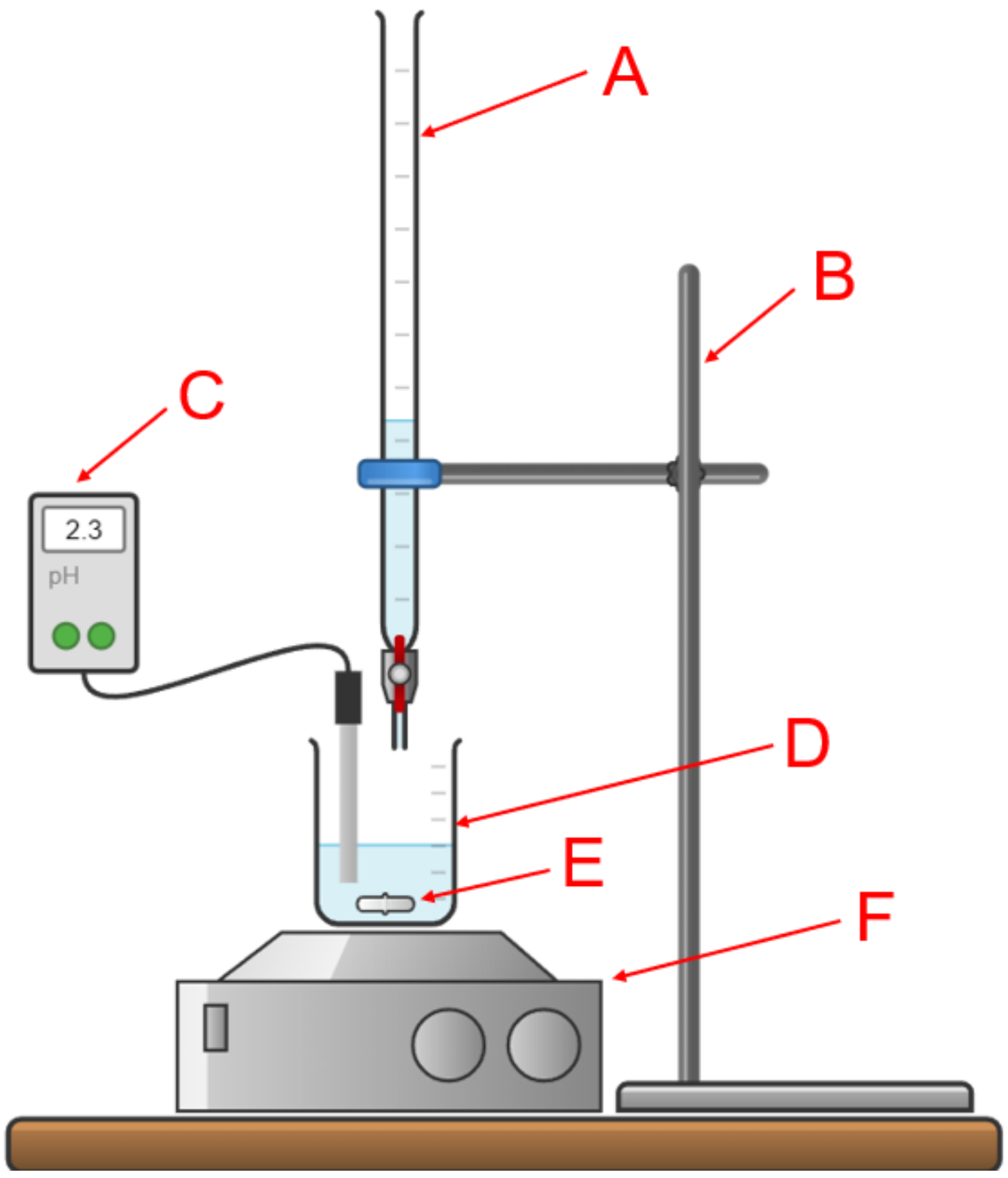

Supplementary Figure 4, Laboratory setup of the neutralisation experiments. A – burette containing seawater, B – clamp stand, C – pH-ORP meter, D – beaker containing river water-seawater mixture, E – magnetic stir bar, F – magnetic stirrer. Figure generated using Chemix.

Triplicates of neutralization experiments using river water collected at Niebla and Gibraleón in April 2024 yield reproducibilities of $Alk_{consumed}$ of 0.9 % and 10.6 % respectively (relative 2 standard deviations), and consistent evolutions of pH with respect to F_{sw} (Supplementary Fig. 5). Changes in pH with respect to F_{sw} also show good agreement between neutralization experiments on river water collected at Niebla and Gibraleón in June 2024 and estuarine distributions measured during this time period.

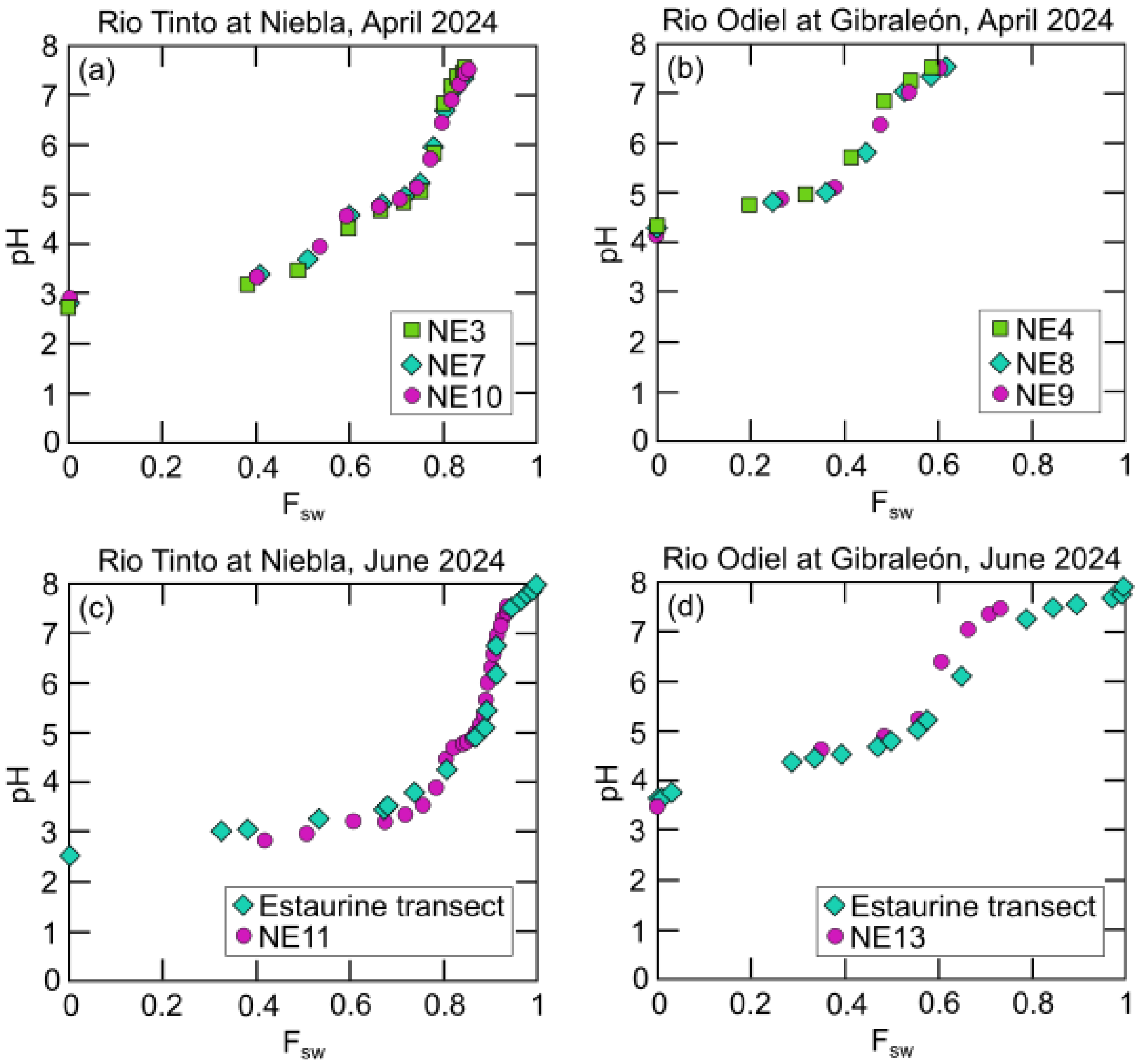

Supplementary Fig. 5, Changes in pH as a function of incremental seawater additions in the neutralization experiments. Panels (a) and (b) show comparisons between triplicate experiments conducted with river water collected at Niebla and Gibraleón respectively in April 2024. Panels (c) and (d) show comparisons between neutralization experiment for river water collected at Niebla and Gibraleón respectively and of the measured estuarine distributions in June 2024. F_{sw} denotes the fraction of the river-water seawater mixture that is composed of seawater.

Four procedural blanks of the neutralization experiments were determined by processing 18.2 MΩ·cm water from a Milli-Q system through the experimental setup in place of AMD impacted river water and seawater. The blanks range between 40 ng and 260 ng for Al, 6 ng and 67 ng for Fe and 3 ng to 14 ng for Mn (Supplementary Table 3). These values represent < 0.24 % of the budgets of these elements in the neutralization experiments, which range between 7 μg to 3600 μg, and are hence deemed negligible.

Supplementary Table 3, Procedure blanks of the neutralization experiments

Blank	Al [ng]	Mn [ng]	Fe [ng]
(1)	94	3	6
(2)	40	8	4
(3)	260	12	67
(4)	163	14	19

***S.7.** Data quality of microwave plasma atomic emission spectroscopy and ion chromatography analyses*

The precision and accuracy of the elemental concentration measurements using microwave plasma atomic emission spectroscopy (MP-AES) was assessed with replicate measurements of the river water Certified Reference Material (CRM) SLRS-6 (Supplementary Table 4). The mean values agree within 5% of the certified values for most elements, with the relative two standard deviations (2RSD) of the replicate measurements typically better than 5%. A notable exception to this is Mn, which features 2RSD's of 22% to 44%. This poorer reproducibility reflects the low concentration of Mn in SLRS-6 (0.039 μM)

which is not representative of the majority of water samples analyzed in this study that feature Mn concentrations ~2 µM to 744 µM.

Supplementary Table 4, Data quality metrics for elemental concentration measurements using microwave plasma atomic emission spectroscopy (MP-AES).

Element	LOD[a]	SLRS-6 certified[b]	SLRS-6 measured[c]	2RSD (%)[d]	n[e]	Accuracy (%)[f]	Measurement batch
Al (µM)	0.46	1.26	1.24	3.3	4	1.1	240927 - Tinto branch
	0.19		1.31	2.8	4	4.1	240930 - Odiel branch
Fe (µM)	0.22	1.51	1.40	10.9	4	7.4	240927 - Tinto branch
	0.23		1.59	2.4	4	4.9	240930 - Odiel branch
Mn (µM)	0.01	0.039	0.038	22	4	2.5	240927 - Tinto branch
	0.01		0.040	44	4	2.5	240930 - Odiel branch
Ca (µM)	0.11	219	192	1.8	4	12.2	240927 - Tinto branch
	0.17		210	3.0	4	3.9	240930 - Odiel branch
Mg (µM)	0.02	88	92	3.1	6	5.0	250430 – both branches
Na (µM)	6.80	120	133	4.8	6	10.7	250430 – both branches
K (µM)	0.12	17	15	1.3	6	11.0	250430 – both branches
Si (µM)	0.88	NA	71.9	1.0	4	NA	240927 - Tinto branch
	0.98		67.1	6.4	4	NA	240930 - Odiel branch

[a] Limit of detection (LOD) defined as 3 times the standard deviation of measurements of the blank 2% HNO_3 carrier acid.

[b] Certified concentrations for the river water Certified Reference Material SLRS-6.

[c] The mean of replicate measurements of SLRS-6 conducted during each measurement batch.

[d] Two times the relative standard deviation (2RSD) in percent of replicate measurements of SLRS-6 conducted during each measurement batch.

[e] The number (n) of replicate measurements of SLRS-6 conducted during the measurement batch.

[f] The relative difference between the certified and measured concentration.

The river water - anions CRM, LGC6025, was analyzed using ion chromatography alongside the samples for dissolved SO_4^{2-} and Cl^-. Measured values agree within 6 to 17% of the certified values with reproducibilities of <5% (relative 2 standard deviations) (Supplementary Table 5).

Supplementary Table 5, Data quality metrics for SO_4^{2-} and Cl^- concentration measurements using ion chromatography.

Species	LOD[a]	LGC6025 certified[b]	LGC6025 measured[c]	2RSD (%)[d]	n[e]	Accuracy (%)[f]	Measurement batch
Cl^- (µM)	0.13	883	795	3.4	8	11	All samples
SO_4^{2-} (µM)	0.001	682	642	NA	1	6	June 2024 samples
	0.16		820	4.6	8	17	April 2024 samples

[a] Limit of detection (LOD) defined as 3 times the standard deviation of measurements of deionized water blanks.

[b] Certified concentrations for the river water Certified Reference Material LGC6025.

[c] The mean of replicate measurements of LGC6025 conducted during each measurement batch.

[d] Two times the relative standard deviation (2RSD) in percent of replicate measurements of LGC6025conducted during each measurement batch.

[e] The number (n) of replicate measurements of LGC6025 conducted during the measurement batch.

[f] The relative difference between the certified and measured concentration.

S.8 *Protocols for Fe speciation measurements using spectrophotometry*

At the University of Huelva, total dissolved Fe concentrations were determined by the addition of 1ml of acetate buffer (5.2M ammonium acetate in an 8.3M acetic acid solution), 2ml of 21mM phenanthroline chloride and 0.8ml of 1.4M hydroxylamine hydrochloride to between 0.2ml and 4ml of unacidified sample. Dissolved concentrations of Fe^{2+} were determined by the addition of 1ml of acetate buffer (5.2M ammonium acetate in an 8.3M acetic acid solution) and 2ml of 21mM phenanthroline chloride to 0.6 to 4ml of unacidified sample. The solutions were diluted by the addition of 10 to 20ml of deionized water to bring them within the methods calibrated range. Total Fe and Fe^{2+} concentrations were then measured using a HACH DR/890 Colorimeter that had been previously calibrated using a set

of standards. These measurements were conducted within 24 hours of sample collection. Only samples with sufficiently high dissolved Fe concentrations were measured by this technique, corresponding to samples from the Rio Tinto branch of the estuary with pH < 4.3.

At the University of St Andrews, Fe^{2+} concentrations were measured with a Thermo Scientific Evolution 220 UV-visible spectrophotometer. 1ml of sample was mixed with 0.5 ml of acetate buffer solution and 0.5 ml of phenanthroline solution. To prepare the acetate buffer solution, 75 g ammonium acetate were dissolved in ca. 45 mL of deionized water, then 150 ml acetic acid were added, and the solution was topped up with deionized water to 500 ml. The phenanthroline solution was prepared by dissolving 0.5 g of 1,10-phenanthrolinium chloride monohydrate in 100 mL of deionized water. The water sample mixed with the reagents was left to sit for 20 min and then analysed at wavelength 510-512 nm. Calibration standards were prepared from pure a Fe ICP-MS standard solution stabilized with 0.5% H_2SO_4 and converted to Fe^{2+} with ascorbic acid. No sulfuric acid was added to the samples as these already contain sulfuric acid naturally.

Measurements of total dissolved Fe using spectrophotometry at the University of Huelva agree well with those conducted by MP-AES (Supplementary Fig. 6). Sample T1 was analyzed for Fe^{2+} using spectrophotometry at both the University of Huelva and the University of St Andrews, with the results agreeing within 4% (110 μM and 106 μM).

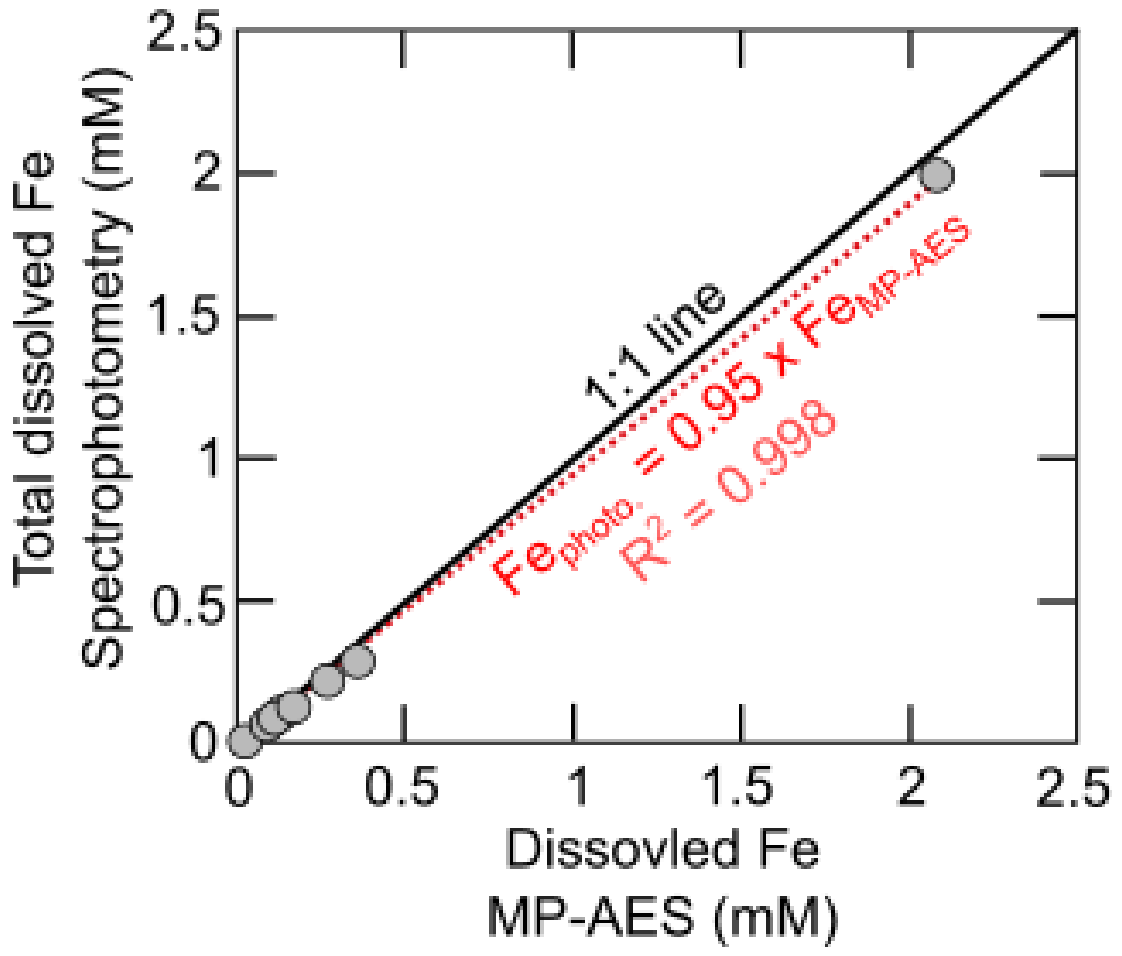

Supplementary Fig. 6, Comparison between total dissolved Fe concentrations determined using spectrophotometry at the University of Huelva and microwave plasma atomic emission spectroscopy (MP-AES).

References

Bigham J. M., Schwertmann, U., Traina S. J., Winland R. L. and Wolf M., 1996, Schwertmannite and the chemical modelling of iron in acid sulfate waters, *Geochimica et Cosmochimica Acta*, 60, 2111-2121.

Kirby C. S. and Cravotta C. A, 2005a, Net alkalinity and net acidity 1: Theoretical considerations, Applied Geochemistry, 20, 1920-1940, doi:10.1016/j.apgeochem.2005.07.002

Kirby C. S. and Cravotta C. A, 2005a, Net alkalinity and net acidity2: Practical considerations, Applied Geochemistry, 20, 1941-1964, doi:10.1016/j.apgepchem.2005.07.003

Millán-Becerro R., Pérez-López R., Cánovas C. R., Macias F. and León R., 2023, Phosphogypsum weathering and implications for pollutant discharge into an estuary, *Journal of Hydrology*, 617, 128943, doi:10.1016/j.jhydrol.2022.128943

Pérez-López R., Millán-Becerro R., Dolores-Basallote M., Carrero S., Parviainen A., Freydier R., Macias F. and Cánovas C. R., 2023, Effects of estuarine water mixing on the mobility of trace elements in acid mine drainage leachates, Marine Pollution Bulletin, 187, 114491, doi:10.1016/j.marpolbul.2022.114491

Simate G. S. and Ndlovu S., 2014, Acid mine drainage: Challenges and opportunities, *Journal of Environmental Chemical Engineering*, 2, 1785-1803, doi:10.1016/j.jece.2014.07.021

Tosca, N. J., McLennan, S. M., Dyar, M. D., Sklute E. C. and Michel F. M., 2008, Fe oxidation processes at Meridiani Planum and implications for secondary Fe mineralogy on Mars. *Journal of Geophysical Research*, 113, doi: 10.1029/2007JE003019.
